# Dispersal of Adult *Culex* Mosquitoes in an Urban West Nile Virus Hotspot: A Mark-Capture Study Incorporating Stable Isotope Enrichment of Natural Larval Habitats

**DOI:** 10.1371/journal.pntd.0002768

**Published:** 2014-03-27

**Authors:** Gabriel L. Hamer, Tavis K. Anderson, Danielle J. Donovan, Jeffrey D. Brawn, Bethany L. Krebs, Allison M. Gardner, Marilyn O. Ruiz, William M. Brown, Uriel D. Kitron, Christina M. Newman, Tony L. Goldberg, Edward D. Walker

**Affiliations:** 1 Department of Microbiology and Molecular Genetics, Michigan State University, East Lansing, Michigan, United States of America; 2 Department of Entomology, Texas A&M University, College Station, Texas, United States of America; 3 Department of Biology, Georgia Southern University, Statesboro, Georgia, United States of America; 4 Department of Entomology, Michigan State University, East Lansing, Michigan, United States of America; 5 Department of Natural Resources and Environmental Sciences, University of Illinois, Urbana, Illinois, United States of America; 6 Department of Entomology, University of Illinois, Urbana, Illinois, United States of America; 7 College of Veterinary Medicine, University of Illinois, Urbana, Illinois, United States of America; 8 Department of Environmental Studies, Emory University, Atlanta, Georgia, United States of America; 9 Department of Pathobiological Sciences, University of Wisconsin, Madison, Wisconsin, United States of America; Mahidol University, Thailand

## Abstract

Dispersal is a critical life history behavior for mosquitoes and is important for the spread of mosquito-borne disease. We implemented the first stable isotope mark-capture study to measure mosquito dispersal, focusing on *Culex pipiens* in southwest suburban Chicago, Illinois, a hotspot of West Nile virus (WNV) transmission. We enriched nine catch basins in 2010 and 2011 with ^15^N-potassium nitrate and detected dispersal of enriched adult females emerging from these catch basins using CDC light and gravid traps to distances as far as 3 km. We detected 12 isotopically enriched pools of mosquitoes out of 2,442 tested during the two years and calculated a mean dispersal distance of 1.15 km and maximum flight range of 2.48 km. According to a logistic distribution function, 90% of the female *Culex* mosquitoes stayed within 3 km of their larval habitat, which corresponds with the distance-limited genetic variation of WNV observed in this study region. This study provides new insights on the dispersal of the most important vector of WNV in the eastern United States and demonstrates the utility of stable isotope enrichment for studying the biology of mosquitoes in other disease systems.

## Author Summary

The distance and direction of adult mosquitoes movement on the landscape are important processes in the spread of mosquito-borne diseases, and are critical to understand to the development of effective intervention programs. Here we present a novel approach to study adult mosquito dispersal by using stable isotope enrichment of natural larval habitats. We apply this technique in a focal hotspot of West Nile virus (WNV) transmission in suburban, Chicago, USA to measure dispersal of *Culex* spp. mosquitoes. We enriched larval mosquitoes in residential catch basins using ^15^N-potassium nitrate and captured adult mosquitoes in traps surrounding these catch basins. Of 10,817 adult female *Culex* mosquitoes trapped and tested for stable isotopes, 12 individuals were enriched with ^15^N, indicating they originated from the catch basins receiving stable isotope amendments. The mean dispersal distance was 1.15 km and maximum flight range was 2.48 km. Ninety percent of the female *Culex* mosquitoes stayed within 3 km of their larval habitat, which corresponds with the distance-limited genetic variation of WNV observed in this study region. This study provides new insights on the dispersal of the most important vector of WNV in the eastern United States and demonstrates the utility of stable isotope enrichment for studying the biology of mosquitoes in other disease systems.

## Introduction

The distance and direction of mosquito movement on the landscape are critical factors in the development of effective strategies for control of both nuisance and vector mosquito species. At small spatial scales, effective mosquito abatement using adult insecticides or larvicides needs to incorporate information on flight range of the intended mosquito target [1]. For example, when controlling *Aedes aegypti*, the vectors of dengue virus, insecticides are sprayed at homes of infected patients and in a specified proximity to the homes based on studies quantifying adult female dispersal [2]–[4]. Short range dispersal of *Ae. aegypti* has been quantified in dengue-endemic areas using genetic markers in relation to habitat structure, in particular presence of road networks, which act as barriers to mosquito dispersal and further influence the local distribution and risk of dengue cases in humans [5], [6]. At large spatial scales, mosquito movement has been implicated in shaping the geographical spread of West Nile virus (WNV) across North America [7], underscoring the importance of vector dispersal for shaping spatial patterns of disease transmission.

Currently, many alternative strategies to insecticides for vector-borne disease control are being implemented, including sterile insect technique, biological control using *Wolbachia*, and genetically modified mosquitoes (reviewed by [8]). For these disease control strategies to succeed and reduce the global burden of vector-borne diseases, a critical parameter necessary for field implementation of these strategies is the distance mosquitoes travel across the landscape. For example, control programs that release sterile, *Wolbachia*-infected, or genetically modified mosquitoes need detailed understanding of flight ranges to determine the appropriate spatial resolution of the release points. Simulation models of these intervention programs often incorporate parameters to represent adult mosquito dispersal [9], [10], although limited data on actual dispersal presents challenges to these models [11]. Given the importance of adult mosquito behavior, mosquito biologists have utilized mark-release-recapture studies for several decades to estimate mosquito dispersal distance and patterns.

Diverse methods have been used to mark mosquitoes to study dispersal including dyes, paints, dusts, trace elements, and radioactive isotopes (reviewed by [1]). The ideal insect marker should persist without inhibiting normal biology, be environmentally safe, cost-effective, and easy to use [12]. However, existing techniques to mark mosquitoes tend to be labor intensive, as they require rearing mosquitoes, marking them in large quantities, and then inspecting large numbers of individuals to detect re-captures [13]. Additionally, the process of rearing adults, marking them, and releasing them may change behavior compared to natural populations [1], [14]. Further, the artificial release of mosquitoes inflates local populations that may contribute to pathogen transmission; this has led to studies where the proboscis has been glued or amputated to prevent feeding [15], a process with potential consequences for mosquito behavior.

In 2008, a meeting of international experts in vector biology discussed critical needs in vector-borne disease control [16]. Among the research priorities highlighted, the panel listed improved technologies for marking insects for studying basic biology. To meet this challenge and to overcome limitations of previous techniques, Hamer et al. [17] developed a stable isotope method to mark naturally-occurring *Culex pipiens*. The laboratory and field experiments from this study suggested life-long marker retention in adults with no apparent impact on survival or body size. Stable isotopes occur naturally in the environment, are non-toxic and non-radioactive, and incorporate into living tissue, which make them safe and useful tracers [18]. Several studies have utilized stable isotopes to study dispersal of adult insects; ^15^N was added to streams, immature aquatic insects incorporated the rare isotope into structural body tissues, and then the emergent adult insects were captured at different distances from the enriched stream [19]–[21]. Additionally, mosquitoes have been enriched with stable isotopes in the context of Sterile Insect Technique programs [22], [23].

Here we report the first application of stable isotopes to study mosquito dispersal in natural field conditions. We enriched naturally existing larval *Culex* mosquitoes with ^15^N in catch basins in Alsip, Illinois and used a large network of traps to capture marked females. This study demonstrates that female *Culex* mosquitoes were capable of flying up to 2.4 km with a logistic distribution function suggesting that 90% of the female *Culex* mosquitoes stayed within 3 km of their larval habitat. We discuss the advantages and disadvantages of the stable isotope enrichment of natural larval habitats and demonstrate how this approach could be a valuable new tool to study dispersal of medically important mosquitoes around the world.

## Methods

### Stable isotope enrichment of catch basins

**Figure 1:**
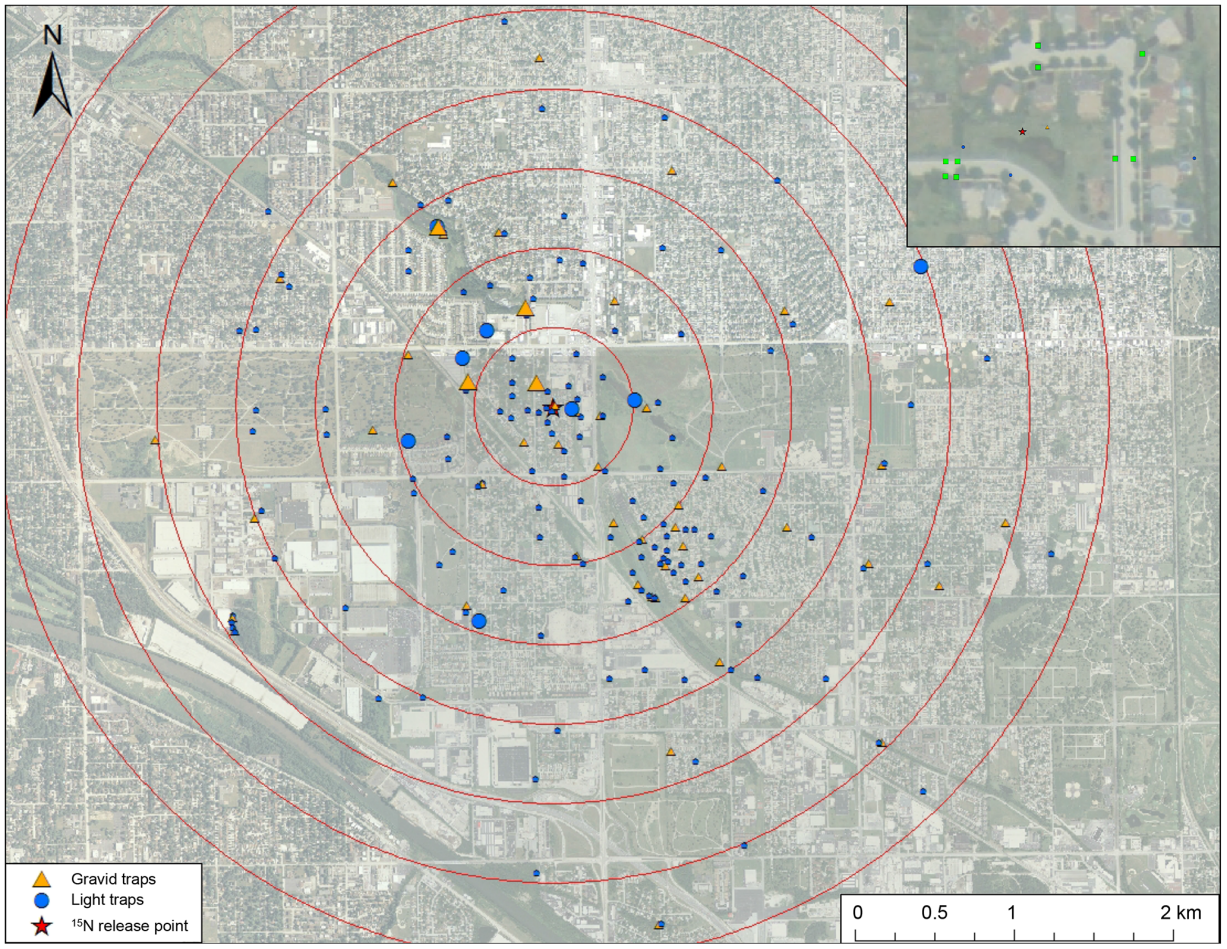
Map of 2010 and 2011 mark-capture study region in suburban Chicago, Illinois. Gravid traps are orange triangles and light traps are blue circles and the larger trap symbols represent the 12 traps that captured marked *Culex* mosquitoes. Not all traps shown where trapped in both years. Red concentric rings represent the seven annuli used to calculate mean distance traveled. Inset shows nine catch basins enriched with ^15^N-potassium nitrate as green squares and the red star represents the mean center of these nine points.

From July to October 2010 and 2011, we treated nine catch basins with ^15^N-potassium nitrate in Alsip, Illinois (41°41′14.56″N; 87°44′32.84″W). The catch basins are stormwater drains designed with a sump to prevent organic debris from entering pipes that lead to an outlet at a creek. The catch basins we treated were the terminal catch basins before water drained through an outlet into a small stream (Stony Creek; Figure 1). The maximum distance between treated catch basins was 153 m. We added stable isotopes with the quantity based on the amount of water present in the catch basin sumps [17]. Briefly, the volume of water in each catch basin sump was estimated under the assumption that sediment comprised 50% of the sump volume. Initial treatment began with a targeted enrichment of 2.0 mg of stable isotope per liter of water in the catch basin. In subsequent treatments that followed rain events, we reduced this amount to a half dose or quarter dose, depending on the amount of flushing that had occurred. In 2010, we delivered ^15^N-potassium nitrate into the 9 catch basins on seven occasions for a total of 38.58 grams being delivered. In 2011, we treated these same catch basins on 14 occasions for a total of 51.36 grams. Fourth instar larvae, pupae, and adult male and female *Culex* mosquitoes were subsampled from these catch basins and submitted for stable isotope analysis to monitor the level of enrichment and adjust the frequency and quantity of isotopic amendment accordingly.

We estimated the production of ^15^N-enriched mosquitoes from the amended catch basins using previously described emergence traps [24]. These traps were necessary because S-methoprene is used by mosquito control agencies in the study region and the presence of larvae or pupae does not necessarily reflect adult emergence. These standardized emergence traps covered a known surface area inside the catch basin and allowed an estimation of the total production of marked adults leaving the catch basin. In 2010 and 2011, we placed and continuously monitored three emergence traps in three of the treated catch basins from July to October. In addition, we estimated the number of *Culex* mosquitoes emerging from all nine catch basins per day from July to October [24].

To ensure that our enrichment activities were not affecting larval mosquitoes outside of our desired areas, we monitored down-stream enrichment by sampling immature mosquitoes and benthic invertebrates (Chironomidae, Amphipoda, Ephemeroptera, Coleoptera) in the Stony Creek; upstream, downstream, and at the outlet opening. We sampled these invertebrates on July 23 and August 31 in 2010 and on August 30 in 2011. Invertebrates from the creek were submitted for stable isotope testing and all δ^15^N values represented natural abundance levels (mean δ^15^N = 11.75) indicating no down-stream enrichment.

We obtained permission to add stable isotopes to the environment by the municipalities, Cook County Department of Public Health, Illinois Department of Public Health, and the Illinois Environmental Protection Agency.

### Adult mosquito trapping

Mosquitoes were trapped from May to October, 2010 and 2011 in Alsip, Blue Island, Chicago, Chicago Ridge, and Oak Lawn, Illinois. We deployed CDC light traps at 100 different locations and gravid traps at 40 locations in 2010 and 83 light trap locations and 33 gravid trap locations in 2011 (Figure 1). The closest mosquito trap was 17.6 m from the centroid of the nine enriched catch basins and the farthest trap was 3.3 km. The mean trap distance from the centroid of the catch basins was 1.47 km and 0.89 km in 2010 and 2011, respectively. These trap locations were distributed in all directions from the catch basins and specific locations were dependent on obtaining permission from landowners. All locations were trapped once per week. Mosquitoes were identified by species and sex, and then pools of up to 50 female *Culex* spp. mosquitoes were tested for WNV using a quantitative RT-PCR [25]. Adult female *Cx. pipiens* and *Cx. restuans* collected in traps were pooled together as *Culex* spp. given the difficulty in distinguishing the two *Culex* species morphologically [26]. RNA was extracted using a MagMAX Viral Total RNA Isolation Kit (Applied Biosystems, Foster City, California). A subset of female *Culex* spp. mosquitoes were placed in pools of up to five individuals and prepared for stable isotope testing as previously described [17]. In 2010 and 2011, we deployed a Hobo weather station (Onset Computer Corporation, Pocasset, MA) placed 1.9 km from the amended catch basins. This station recorded hourly temperature, wind speed, wind direction, and precipitation. We calculated the average wind direction as a combined vector of the mean wind angle and speed [27]. The direction and speed were converted into N-S and E-W components and averaged over the July to September period. The average wind direction in 2010 was 216° (southwest) with a net speed of 1.9 kph and in 2011 was 170° (south) with a net speed of 0.66 kph.

### Stable isotope analysis

All 4^th^ instar larvae, pupae, adult mosquitoes, and other aquatic invertebrates were stored at −20°C and processed for stable isotope analysis as previously described [17]. Briefly, samples were dried at 50°C for 24 h, encapsulated into tin capsules to create a sphere, arranged into a 96-well plate, and submitted for stable isotope analysis at the University of California-Davis Stable Isotope Facility using a PDZ Europa ANCA-GSL elemental analyzer interfaced to a PDZ Europa 20Ð20 isotope ratio mass spectrometer (IRMS; Sercon Ltd., Cheshire, United Kingdom). Additional analyses, that required short turn-around of results in order to guide field enrichment activities, were performed by Isotech Laboratories Inc. by using a Carlo Erba CHNS-O EA1108 (CE Instruments, Milan, Italy) coupled to a ThermoFisher Delta V Plus IRMS (Thermo Fisher ScientiÞc, Bremen, Germany) via a ThermoFinnigan ConFlo III interface (Thermo Electron Corp., Waltham, MA).

### Statistical analysis

Mosquito pools collected in the field and tested for stable isotopes were considered enriched if δ^15^N values were at least three standard deviations above the natural isotopic abundance of *Culex* mosquitoes in our study region [28]. We calculated the mean distance traveled (MDT) and incorporated a correction factor for each annulus given unequal trap density [29]–[32]. We calculated MDT for each year based on the equations in [33].
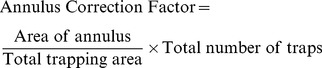



The estimated recaptures (ER) for each annulus were calculated as:
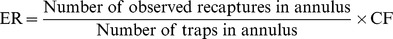


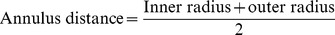
The mean distance traveled (MDT) was calculated as:
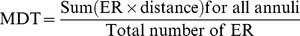



To estimate the probability of detecting a marked mosquito at different distances from the release point, we used a logistic regression in Program R [34]. Similar dispersion models have been used for relating the distance insects travel from a central point [35]. The data from two years were merged and each trap location (n = 167) was given a 1 for detecting a marked individual or a zero for no marked individuals. We used the centroid of the 9 catch basins receiving stable isotopes to calculate the distance from each light or gravid trap. The logistic distribution function of plogis (x) = (1+tanh (x/2))/2 was used for the predictions of detecting a marked mosquito at different distances from the release point.

## Results

### Stable isotope enrichment of catch basins

During the enrichment of nine catch basins receiving ^15^N-potassium nitrate in 2010 and 2011, a subsample of 4^th^ instar larvae and pupae were collected and all were identified as *Culex pipiens* or *Culex restuans*. These immature specimens collected directly from the treated catch basins had a mean δ^15^N of 484.1±73.1 (n = 73) while immature *Culex pipiens* collected from nearby untreated catch basins had a mean δ^15^N of 4.7±0.74 (n = 15).

**Figure 2:**
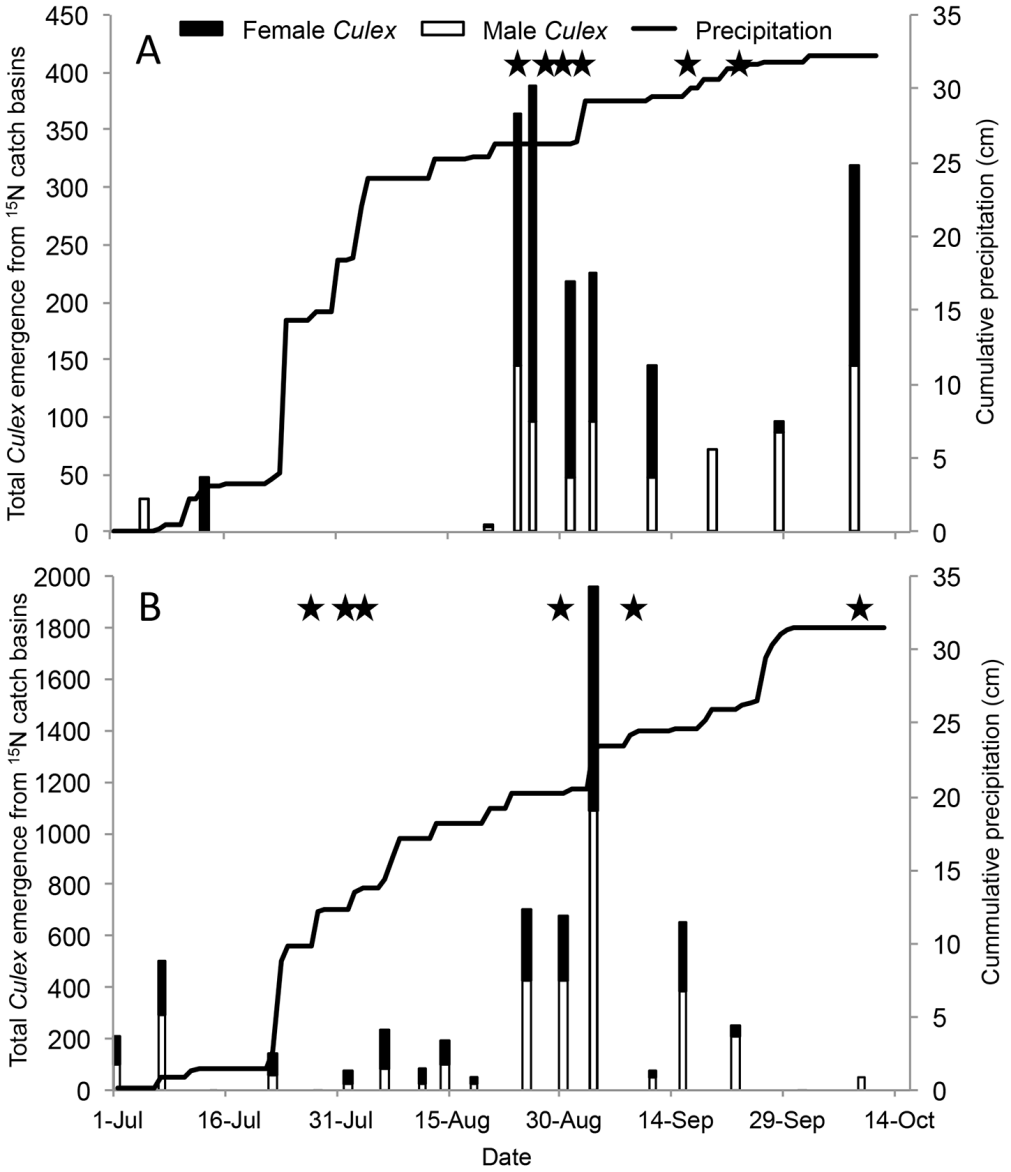
Emergence of *Culex* spp. mosquitoes from the nine catch basins receiving ^15^N enrichment in suburban Chicago in 2010 (A) and 2011 (B). Stars represent the dates when ^15^N-enriched female *Culex* pools were captured in traps.

Using emergence traps, we estimated that the nine enriched catch basins produced 1,138 female and 769 male *Culex* spp. mosquitoes in 2010 and 2,624 female and 3,362 male *Culex* spp. mosquitoes in 2011 from July to October (Figure 2). The emergence of adults from the catch basins declined following large rain events (e.g. greater than 1 cm per day [24]) and the capture of marked pools from traps tended to occur following increased periods of emergence. A total of 343 larvae collected in catch basins or adults collected in emergence traps were identified to species during the two years and 333 (97%) were *Cx. pipiens* and 10 (3%) were *Cx. restuans*.

### Adult mosquito trapping

In 2010, we collected 271,594 female mosquitoes of which 30,261 were *Culex* spp. mosquitoes (11.1%). Of the 2,255 *Culex* spp. mosquito pools (23,068 individuals) tested for WNV, 166 pools were positive with a peak infection rate of 42.6 per 1000 individuals (95% CI of 27.9–63.3) at the end of August. In 2011, we collected 227,036 individual mosquitoes of which 15,263 were *Culex* spp. mosquitoes (6.7%). Of the 1,954 *Culex* spp. mosquito pools (11,639 individuals) tested for WNV, 6 pools were positive with a peak infection rate of 2.31 per 1000 individuals (95% CI of 0.4–7.6) occurring in mid-August.

In 2010, 1,529 female *Culex* spp. mosquito pools (7,193 individuals) were collected and tested for stable isotopes and 6 pools were enriched (Figure 1). In 2011, 913 female *Culex* spp. mosquito pools (3,624 individuals) were collected and tested for stable isotopes and 6 pools were enriched. The 12 marked pools had a mean δ^15^N of 285.4±198.4. The mean δ^15^N of all un-enriched female *Culex* spp. mosquito pools was 6.6±0.04. Based on the estimated number of enriched female *Culex* mosquitoes emerging from the treated catch basins, we obtained a re-capture rate of 0.52% in 2010 and 0.23% in 2011, under the simplifying assumption that marked pools contained only one marked individual. The MDT for 2010 was 1.44 km and 2011 was 0.86 km. The closest trap containing a marked mosquito was 123.9 m from the release point and the farthest was 2.48 km (mean = 0.9 km, S.E. = 0.19). The marked female mosquito captured at 2.48 km occurred on September 22, 2010 in a trap with a 68 degree bearing from the release point. During the previous night before this female was captured (8pm to 8am) there was a mean wind speed of 3.3 km per hour (gusts up to 16.7 km per hour) with a mean bearing of 254 degrees (WSW at 74 degrees), which is in the direction of the trap that captured the marked female mosquito.

**Figure 3:**
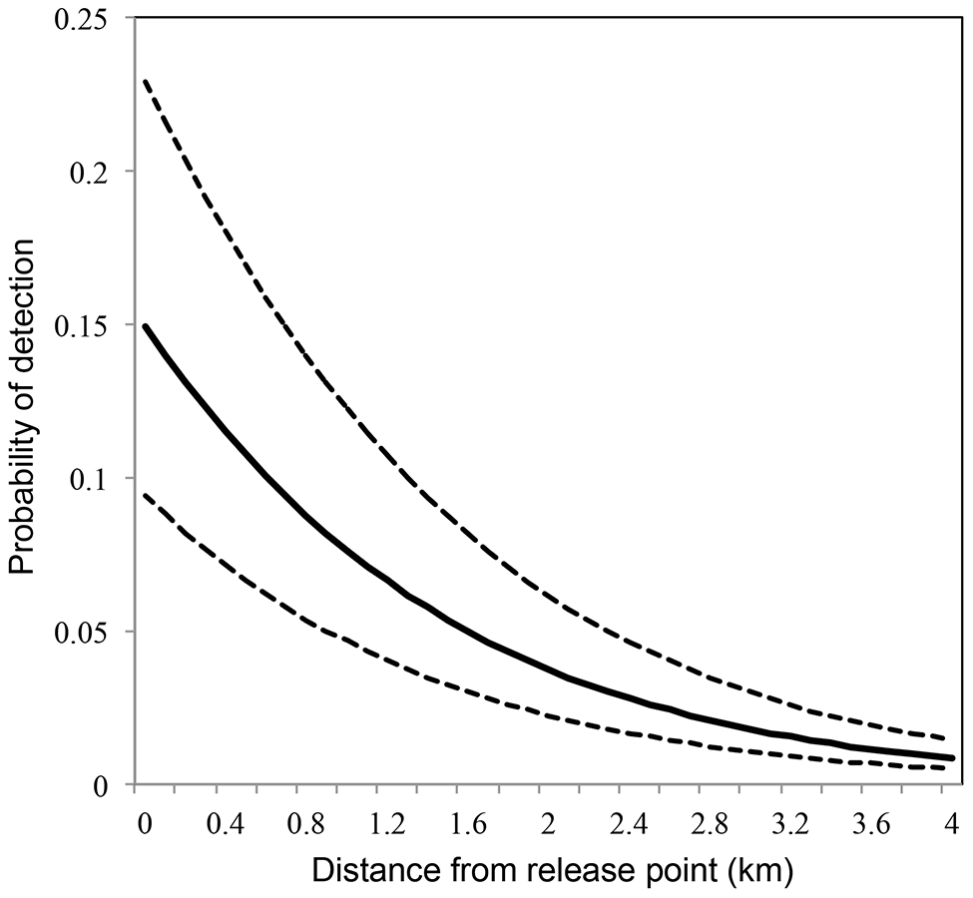
Probability of detecting a marked female *Culex* spp. mosquito at different distances from larval origin. The prediction function is equal to (1+tanh((−0.76*x – 1.74)/2))/2. Dashed lines represent standard error.

For the two years combined, the probability of detecting a marked mosquito at different distances from the release point was estimated using a logistic distribution function of y = (1+tanh((−0.76*x – 1.74)/2))/2 (Figure 3). Based on this model, 80% of the marked mosquitoes stayed within 2.1 km of the release point and 90% stayed within 3 km.

## Discussion

This study used stable isotope enrichment to measure the dispersal of *Culex* spp. mosquitoes in an urban hot spot of WNV transmission. The majority of the dispersal studies of *Culex* mosquitoes to date have focused on *Cx. quinquefasciatus* or *Cx. tarsalis* (reviewed by [1]) and there are very few dispersal studies for *Cx. pipiens* in the U.S. Given the importance of *Cx. pipiens* as a primary enzootic vector of WNV in the eastern half of the U.S. north of 36° latitude [36], [37], the paucity of data is unfortunate. One exception is a mark-release-recapture study by Jones et al. [38] conducted on *Cx. pipiens*, using fluorescent dust near Washington, DC. However, this study was primarily designed to study survival. A second study by Ciota et al. [39] analyzed dispersal of *Cx. pipiens* in New York using a novel labeling approach that allowed mosquitoes emerging from natural container habitats to self-mark with fluorescent dust. Ciota et al. [39] report a MDT of 1.37 km for *Cx. pipiens* (maximum flight range of 1.98 km) and the current study reports a MDT of 1.15 km (maximum flight range of 2.48 km), suggesting similar estimates between the two studies. Given the low detection probability of capturing a marked mosquito at large distances from the point of origin, these studies emphasize the potential for female *Cx. pipiens* to travel several kilometers from larval habitats of origin. However, the current dispersal estimates should be cautiously interpreted given the limited capture of marked female *Cx. pipiens* mosquitoes in the Ciota et al. [39] study (n = 10) and in the current study (n = 12). Future studies with designs that capture more marked mosquitoes will reduce the uncertainty of such estimates.

The mosquito dispersal documented in the current study is of direct relevance to the enzootic transmission of WNV and “spillover” to humans. From 2002 to 2012, the study region defined by the seven annuli contained the geocoded addresses of 57 human cases of WNV (Illinois Department of Public Health). *Culex* emerging from catch basins represent the same population that are part of the enzootic cycle feeding on birds [25] and are very likely also responsible for the bridge transmission to humans [40]. The study region has a radius of 3.5 km and includes 8 municipalities (Alsip, Oak Lawn, Chicago Ridge, Worth, Chicago, Palos Heights, Blue Island, and Midlothian), in which mosquito control efforts vary considerably. Our estimates that nearly 20% of *Culex* mosquitoes were able to travel over 2 km from their larval environment demonstrates that the mosquito control efficacy in one small municipality can affect the level of WNV transmission and of risk of human exposure in adjacent regions. Moreover, Bertolotti et al. [41] studied the fine-scale genetic variation of WNV in this suburban Chicago study region and found significant negative spatial autocorrelation at distances beyond 4 km. This evidence of distance-limited viral transmission corresponds well with the distance female *Culex* mosquitoes moved in the current study.

The stable isotope marking technique offers advantages and disadvantages over traditional mosquito dispersal studies. The ability to mark wild mosquitoes in natural containers with a non-invasive marker is an ideal approach to avoid artifacts of the marker or unnatural larval diet that may influence dispersal behavior [1], [12]. Additionally, our previous laboratory experiment revealed low decay rates of ^15^N in mosquitoes held for 55 days post-emergence [17]. Based on the enrichment achieved in the field during the current study, the stable isotope marker should offer sufficient retention for the life of the mosquito, which is fortunate given the significance of old females for disease transmission. The advantage of the larval site label also comes with the disadvantage of not being able to control how many marked mosquitoes emerge. In the current study, the relatively wet summers of 2010 and 2011 compromised this study given that the rain events washed the water and larvae out of the catch basins (Fig. 2; [24], [42]. Additionally, local mosquito control efforts used S-methoprene based products to treat mosquitoes in the same catch basins we were enriching with stable isotopes, although the immediate effect on the study was not quantified.

Another challenge of the larval site labeling approach is that the release of marked mosquitoes is over a prolonged time period and is thus not a defined release event. This confounds the effort to determine the age of the captured marked mosquitoes. Although this study estimated the number of marked mosquitoes emerging from the treated catch basins, the uncertainty associated with this estimate limits the ability to estimate the size of the adult mosquito population [1]. Another factor to consider with isotopic enrichment of mosquito larval environments in the field is the potential for downstream enrichment. In our case, we received many rain events during the study period, so dissolved potassium nitrate or microbes enriched with ^15^N would have washed into the Stony Creek at the catch basin outlets. However, we monitored aquatic invertebrates in these downstream areas and none were enriched, likely indicating that the large volume of water in the stream diluted the ^15^N to a concentration that was unable to bioaccumulate into the food chain and into invertebrates measurably.

The stable isotope mark-capture study offers a unique perspective on mosquito dispersal and should broadly be useful for other mosquito-borne disease systems. *Aedes aegypti*, responsible for an estimated 50–100 million annual human cases of dengue virus [43], is a container-breeding mosquito ideally suited for applying this stable isotope mark-capture study. Although *Ae. aegypti* is generally characterized as having limited dispersal [1], [44], the unique ability of life-long marker retention might yield a unique perspective on *Ae. aegypti* movement. This is especially important given current techniques of gene-driving and *Wolbachia*-induced population suppression or reduced vector competence aimed at the global elimination of dengue virus [8]. Understanding the distance between *Anopheles* spp. larval habitat and human exposure to malaria could improve control programs and mitigate disease transmission. Importantly, the enrichment of larval mosquito environments with stable isotopes is relatively inexpensive and easy to implement. Adult mosquitoes captured in traps need to be kept frozen or dried prior to the stable isotope analysis, either of which would be possible in remote field locations with limited facilities. Besides the labor and consumables to run mosquito traps, the most expensive aspect of this kind of project is the stable isotope analysis. Our project collected 2,442 pools between the two years that were tested at $8 per sample totaling $19,536. Different stable isotope facilities charge variable amounts, and the cost per sample is dropping [18]. Given the ability to measure mosquito dispersion, including epidemiologically important old females, this tool should be useful for studying the dispersal behavior of other medically important arthropods.
